# Maternal and infant oral health benefits from mothers receiving prenatal total oral rehabilitation: a pilot prospective birth cohort study

**DOI:** 10.3389/froh.2024.1443337

**Published:** 2024-08-13

**Authors:** Ruqian Yang, Noha Rashwan, Nisreen Al Jallad, Yan Wu, Xingyi Lu, TongTong Wu, Jin Xiao

**Affiliations:** ^1^ Eastman Institute for Oral Health, University of Rochester Medical Center, Rochester, NY, United States; ^2^ Department of Biostatistics, University of Rochester Medical Center, Rochester, NY, United States

**Keywords:** prenatal oral healthcare, pregnant women, cariogenic microorganism, early-life dental care, dental caries

## Abstract

**Aim:**

This study aimed to evaluate the maternal and infant oral health benefits from mothers receiving prenatal total oral rehabilitation (PTOR) before childbirth.

**Methods:**

Building upon our previous investigation, in which 15 expectant mothers received PTOR before their third trimester, achieving a state of oral health free from disease prior to delivery, we conducted a follow-up study to monitor these mothers and their newborns until they reached 2 years of age. We assessed the impact of PTOR on maternal and infant oral health, the utilization of dental care during the postpartum/early-life period, and the carriage of oral cariogenic microorganisms among mothers and their infants. Control groups consisting of 11 children and 17 mothers who did not undergo PTOR were included for comparative analysis.

**Results:**

PTOR demonstrated a sustained improvement in maternal oral health outcomes by the end of 2 years postpartum, evidenced by a reduction in the Plaque Index and decayed surfaces compared with the control group (*p* < 0.05). PTOR was also associated with increased perinatal oral health literacy compared with the baseline of the mothers themselves (*p* < 0.05). In addition, PTOR led to a notable increase in maternal dental care utilization, rising from 26.7% before PTOR to 80% at 1 year postpartum and 70% at 2 years postpartum. Intriguingly, 40% of infants in the PTOR group had their first dental visit before reaching 1 year of age, in contrast to national data from the USA indicating a rate of less than 1%. Furthermore, a decrease in plaque *Streptococcus mutans* was observed in PTOR mothers 2 years postpartum, compared with both their baseline carriage and that of the control group (*p* < 0.05). Infants in the PTOR group also had a lower incidence of early childhood caries, with 18% in the PTOR group vs. 27% in the control group, although this difference was not statistically significant due to the small sample size.

**Conclusions:**

PTOR is associated with sustained oral health benefits and improves dental care utilization by mothers and their infants. Large-scale clinical trials are warranted to validate these study findings.

## 1 Introduction

Over time, considerable interest has been paid to the impact of pregnancy and the postpartum period on oral health. Pregnancy induces various physiological changes in the body, primarily through hormonal changes that amplify the inflammatory response, consequently impacting the gingival and periodontal tissue (1–4). In addition, pregnant women are more susceptible to dental caries due to factors such as vomiting, increased cravings for sugary snacks, and a diminished focus on oral hygiene because of their physical condition. Furthermore, salivary *Streptococcus mutans* counts significantly increase in the second and third trimesters and postpartum (5), and mean pH and mean salivary total calcium content decrease in the third trimester and postpartum (1, 3). Despite conflicting data, literature has revealed a potential positive association between periodontal disease and adverse pregnancy outcomes, such as preterm birth and low birth weight infants, as well as cardiovascular disease, type II diabetes, kidney disease, and respiratory disease (4, 6, 7).

Maintaining good oral health during pregnancy, infancy, and childhood is crucial. However, insufficient education is provided to pregnant women, parents, and infant caregivers regarding preventive oral care practices such as fluoride use and dietary habits (8). This lack of education contributes to a significant public health issue with potential intergenerational health implications, including negative impacts on children's oral health (9). Early childhood caries (ECC), in particular, is recognized as the most prevalent chronic oral disease among children, accounting for nearly 1.8 billion new cases per year globally (10).

ECC is defined as the presence of at least one decayed, missing, or filled tooth surface in a child who is 71 months of age or younger (11). ECC affects approximately 37% of children in the USA between the ages of 2 and 5 (10, 12) and up to 73% of preschoolers from socioeconomically disadvantaged backgrounds in both developing and developed nations (13). The prevalence rate of ECC is five times higher than that of the next most common condition, asthma, which affects 60%–90% of children worldwide (14, 15). ECC is a multifactorial disease influenced by genetic, behavioral, environmental, dietary, and microbial factors (16). ECC imposes significant burdens on children, families, and the healthcare system. Children with ECC often experience difficulties with eating, disrupted sleeping, orofacial pain, emergency room visits, hospitalization, and in severe cases, even death (12, 17). Moreover, ECC is associated with an increased risk for caries in permanent dentition (18).

Pregnancy is the ideal time for promoting ECC prevention (13, 19). Poor maternal oral health increases the risk of cariogenic microorganisms being transmitted from mother to child, thus elevating the likelihood of developing caries in children (10, 20). Previous studies have shown that mothers’ oral health and behaviors have a substantial impact on their children's oral health and access to dental care (13). However, existing literature lacks evidence regarding the health advantages and oral microbial alterations resulting from dental restorative treatment for caries control during pregnancy. Furthermore, the extent to which such interventions prevent ECC remains uncertain. In our previous study of prenatal total oral rehabilitation (PTOR), we focused on the critical prenatal period and aimed to restore women's oral health to a “disease-free status” before childbirth (9). PTOR encompassed a comprehensive examination, dental cleaning, and necessary procedures such as periodontal treatment, restorative procedures for dental caries, root canal therapy, and extractions for non-restorable teeth. Our findings revealed improved oral health and increased perinatal oral health literacy in mothers, as well as reduced *S. mutans* oral carriage and alterations in the oral microbiome within a 2 month follow-up during pregnancy (9, 21). In the current study, we have extended the follow-up period for mothers and their newborns after childbirth. Our objective is to evaluate the impacts of PTOR on the oral health of mothers and infants, their utilization of dental care services, and the carriage of cariogenic microorganisms during the initial 2 years of the children's lives.

## 2 Materials and methods

### 2.1 Participants and eligibility

In our previous investigation, 15 underserved pregnant women underwent PTOR before entering their third trimester of pregnancy at the Perinatal Dental Clinic of the University of Rochester Medical Center (URMC) Eastman Institute for Oral Health (EIOH). PTOR encompasses the comprehensive approach mentioned above and elsewhere (9, 21). In the current study, 11 out of the initial 15 women who received PTOR continued their follow-up at 1 year and 2 years postpartum. In addition, after delivery, their infants (*n* = 11) were enrolled in this study and monitored at the ages of 1 and 2 years. A group of children (*n* = 22) and mothers (*n* = 17) matching the PTOR mother–child dyads were included as the control group. All participants in both the PTOR and control groups were from low-income families, determined by their income levels: 138% of the Federal Poverty Line and eligible for New York state-supported medical insurance. This study was approved by the University of Rochester Research Subject Review Board (#4628 and #1248). All participants provided written consent before engaging in the study activities.

The exclusion criteria were as follows: (1) having a decisional impairment, rendering them incapable of making an informed decision; (2) received oral and/or systemic antifungal therapy within 90 days of the baseline study visit; (3) requiring premedication before dental treatment; (4) having more than eight missing teeth, excluding third molars and orthodontically extracted teeth; (5) using removable dental prosthesis for restoring missing teeth; (6) having an orofacial deformity or tumor (e.g., cleft lip/palate); and (7) having a severe systemic disease (e.g., HIV infection). Mothers in the control group fulfilled all inclusion and exclusion criteria, except they were enrolled during their postpartum years spanning from 3 to 5 years.

The inclusion criteria for PTOR children were as follows: (1) mother enrolled in this study and (2) a male or female child younger than 3 years of age. The exclusion criteria were (1) having an orofacial deformity (cleft lip, cleft palate, or oral-pharyngeal mass) and/or (2) a severe systematic disease, e.g., Down syndrome. The control group children fulfilled all inclusion and exclusion criteria.

### 2.2 Data collection and comprehensive oral examination

Demographic and socioeconomic information, oral hygiene practices, and dental care utilization were obtained via questionnaires (Supplementary Material 1, 2). Medical history and medication details were self-reported and cross-verified with electronic medical records. Perinatal oral healthcare literacy scores (ranging from 0 to 7) were collected using questionnaires. At each study visit, participants independently completed a questionnaire containing seven true/false questions. These questions were designed to assess the mothers’ knowledge of oral health, specifically focusing on oral hygiene practices and the prevention of early childhood caries (Supplementary Material 1). Orofacial pain was assessed using the Numeric Rating Scale (NRS) score on a scale of 0–10, with 10 indicating unbearable pain. Birth weight *Z*-scores of infants were calculated according to the WHO growth standard for full-term infants and Fenton 2013 growth calculator for preterm infants (22).

Comprehensive oral examination was conducted by one of two calibrated dentists in a dedicated examination room at the URMC, using standard equipment. Dental plaque was assessed using the Plaque Index (PI) according to Löe and Silness (23), with each of the four gingival areas of the tooth being assigned a score from 0 to 3. Dental caries was assessed using DMFT/*dmft* (decayed, missing, and filled teeth) and the International Caries Detection and Assessment System (ICDAS) criteria. Bleeding on probing (BOP) for the mothers was evaluated using a standard UNC15 periodontal probe. Inter- and intra-examiner agreements for the evaluated criteria were calculated using Kappa statistics, surpassing 90% during the calibration process.

### 2.3 Oral sample collection and processing

Saliva and plaque samples were collected using methods described previously (9). Study subjects were instructed to refrain from eating, drinking, or brushing their teeth for 2 h before providing the oral samples. Approximately 5 ml of unstimulated whole saliva samples were collected by participants spitting into a sterile 50 ml centrifuge tube. Supragingival plaques from the entire dentition were obtained using a sterilized periodontal scaler and then resuspended in 1 ml of a 0.9% sodium chloride solution in a sterilized Eppendorf tube. Saliva and plaque were kept on ice and transported to the laboratory for processing within a 2 h time frame. Saliva and plaque samples underwent a gentle vortexing and sonication process to break down any aggregation. The sonication cycle was repeated three times, with each cycle of 10 s of sonication followed by a 30 s rest on ice. BBL™ CHROMagar™ Candida (BD, Sparks, MD, USA) was used for *Candida* spp isolation and Mitis Salivarius with Bacitracin selective medium was used for *S. mutans* isolation. *Candida* spp. and *S. mutans* were incubated at 37°C with 5% CO_2_ for 48 h. *Candida* spp. and *S. mutans* colonies were quantified using colony-forming units (CFUs).

### 2.4 Statistical analysis

Propensity scores were employed to select the control group from two existing historical cohorts. The control group of mothers was selected from a cohort consisting of 40 postpartum women, with age being the matching parameter. The control group of children was selected from a cohort consisting of 160 infants, with matching parameters including age, ethnicity, and race. The characteristics of the PTOR and control groups were compared using statistical methods appropriate for the nature of the data. *T*-tests or Mann–Whitney *U* tests were used for continuous data, depending on the normality of the data. For categorical data, such as demographic characteristics, chi-square or Fisher's exact tests were used. To assess changes in oral health conditions (BOP, orofacial pain score, and perinatal oral health literacy), paired t analysis or Wilcoxon signed ranks for continuous data, and the McNemar test for categorical data, were conducted. Regarding the carriage of *Candida albicans* and *S. mutans*, the CFU values were converted to natural log values for subsequent statistical analysis. All statistical tests were conducted with a two-sided significance level of 5%. Propensity score matching the control to the PTOR isolation was implemented using R, version 4.2.2 (R Foundation for Statistical Computing, Vienna, Austria). Other statistical analyses were conducted using SPSS, version 28.0 (IBM Corp., Armonk, NY, USA).

## 3 Results

Among the 11 mother–child dyads, 10 completed 1- and 2-year postpartum visits. Statistical analysis revealed no significant differences between the PTOR mothers and control group mothers in terms of age, ethnicity, medical conditions, and educational levels (*p* > 0.05) (Table 1). Similarly, no statistical differences were observed between the PTOR children and the matched control group regarding the children's demographic and medical conditions (*p* > 0.05).

**Table 1:** Demographic medical characteristics of the PTOR participants.

Categories	Treatment (*n* = 11)	Control (*n* = 17)	*p*-value
Mothers			
Age (year)	30.3 ± 7.5	31.4 ± 6.9	0.68
Race			
Black	63.6% (7)	17.6% (3)	0.05
White	27.3% (3)	64.7% (11)	
Other	9.1% (1)	17.6% (3)	
Ethnicity			
Hispanic	9.1% (1)	5.9% (1)	0.75
Non-Hispanic	90.9% (10)	94.1% (16)	
Diabetes mellitus (yes)	9.1% (1)	0% (0)	0.21
Asthma (yes)	18.2% (2)	5.9% (1)	0.30
Hypertension (yes)	90.9% (10)	82.4% (14)	0.53
Smoking (yes)	9.1% (1)	29.4% (5)	0.20
	Treatment (*n* = 11)	Control (*n* = 22)	*p*-value
Children			
Gender			
Female (yes)	63.6% (7)	45.5% (10)	0.33
Race			
Black	63.6% (7)	68.2% (15)	0.96
White	27.3% (3)	22.7% (5)	
Other	9.1% (1)	9.1% (2)	
Ethnicity			
Hispanic	18.2% (2)	27.3% (6)	0.57
Non-Hispanic	81.8% (9)	72.7% (16)	

### 3.1 Maternal *S. mutans* and *C. albicans* carriage in the postpartum period

**Figure 1:**
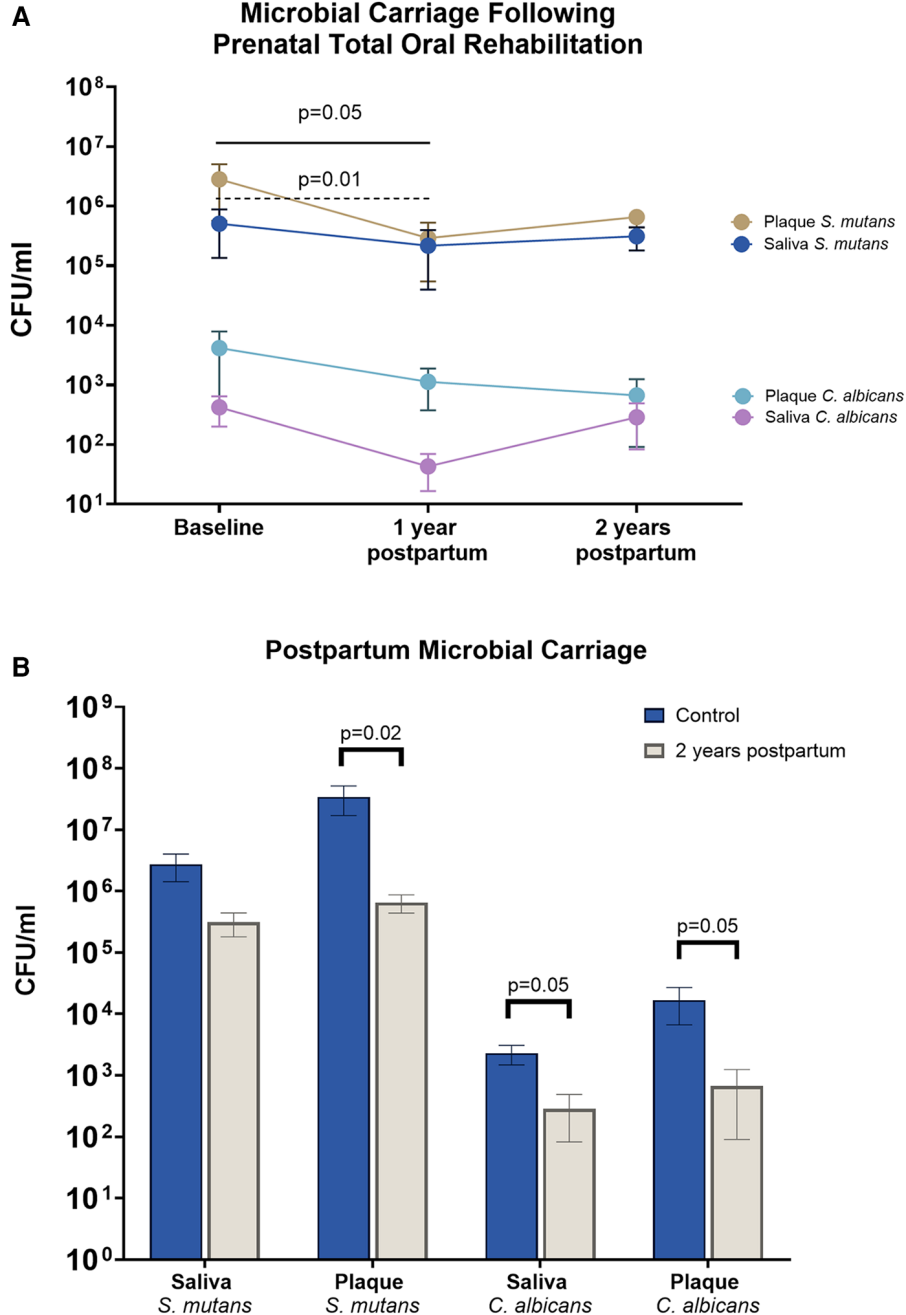
Mother's microbial carriage upon receiving PTOR. (**A**) *Streptococcus mutans* and *Candida albicans* in the saliva and plaque of PTOR mothers at 1 and 2 years postpartum. Salivary and plaque *S. mutans* decreased at 1 year postpartum compared with the baseline visit (*p* = 0.05 in saliva and *p* = 0.01 in plaque). Although salivary and plaque *S. mutans* remained decreased at 2 years postpartum, the change was not statistically significant. Following PTOR, a trend of reduction was noted in both salivary and plaque *C. albicans*; however, no statistically significant difference was found between the baseline and follow-up visits. (**B**) Comparison between PTOR mothers at 2 years postpartum and a control group of mothers of preschool children. A significant decrease was observed in the amount of plaque *S. mutans* (*p* = 0.02), salivary *C. albicans* (*p* = 0.05), and plaque *C. albicans* (*p* = 0.05) among PTOR mothers 2 years after childbirth, compared with mothers in the control group.

Longitudinal changes in salivary and plaque *S. mutans* and *C. albicans* at 1 and 2 years postpartum are shown in Figure 1A. A significant reduction in salivary and plaque *S. mutans* was observed at 1-year postpartum compared with the baseline (*p* < 0.05). Despite salivary and plaque levels of *C. albicans* showing a decreasing trend up to 2 years postpartum, no statistically significant differences were observed (*p* > 0.05).

Compared with the control group, there was less *S. mutans* carriage in plaque in the PTOR group (*p *< 0.05). The *C. albicans* carriage in saliva and plaque in the PTOR group at the 2-year postpartum visit was also lower than that of the control group (*p* < 0.05).

### 3.2 Maternal oral health conditions, knowledge, and dental utilization

**Figure 2:**
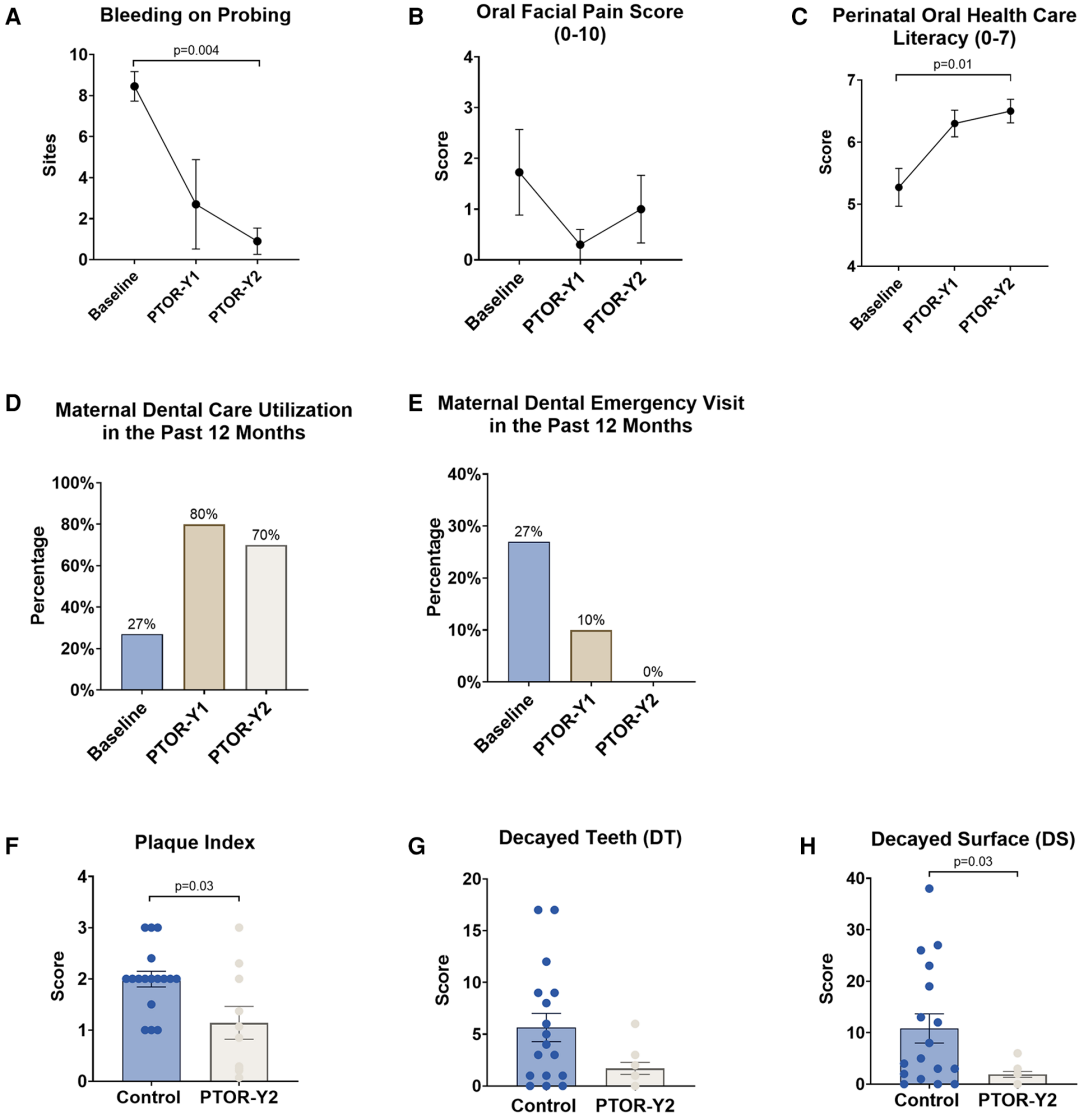
Changes in mother's oral health conditions, literacy, and dental utilization upon receiving PTOR. (**A**) The periodontal condition, as indicated by BOP, showed a significant improvement following PTOR, with a statistical difference between the baseline and 2 years postpartum (*p* = 0.004). (**B**) Orofacial pain scores, ranging from 0 to 10, exhibited a trend of reduction; however, there was no statistically significant difference between time points. (**C**) Perinatal oral healthcare literacy (maximum score of 7) continued to increase upon receiving PTOR, with statistical significance between the baseline and 2 years postpartum (*p* = 0.01). (**D**) A significant improvement in dental utilization of the past 12 months was observed 1 and 2 years postpartum among women who received PTOR. (**E**) Women in the PTOR group had a reduced incidence of dental emergencies 1 year postpartum and no noted emergencies 2 years postpartum. (**F**) Compared with the control group, mothers who received PTOR had better oral hygiene practices, as reflected by a significant reduction in the Plaque Index (*p* = 0.03). (**G**) Decayed teeth numbers and (**H**) decayed tooth surfaces decreased in the PTOR mother group compared with the control group, and the difference in decayed surfaces was statistically significant (*p* = 0.03).

At the 2-year postpartum visit, PTOR mothers exhibited a significant improvement in their periodontal condition, reflected by a reduction in BOP compared with their baseline before receiving PTOR (*p* < 0.05), as shown in Figure 2. Specifically, the average number of BOP sites decreased from 8 at baseline to 1.1 at 2 years postpartum (Figure 2A). Notably, no increase in orofacial pain was observed following PTOR; instead, a downward trend in reduced orofacial pain scores was noted after mothers underwent PTOR (Figure 2B).

Furthermore, significant increases in the utilization of routine dental care were observed post-PTOR, with rates increasing from 27% at baseline to 80% at 1 year and 70% at 2 years postpartum (Figure 2D). This increase in routine dental care utilization was associated with a reduction in emergency care usage. PTOR resulted in decreased utilization of emergency dental care for PTOR mothers, with a utilization rate decreasing from 27% before PTOR to 10% at 1 year postpartum and reaching zero utilization at 2 years postpartum (Figure 2E).

Moreover, PTOR mothers demonstrated a significant improvement in perinatal oral healthcare literacy, a benefit that persisted up to 2 years postpartum (Figure 2C). Compared with mothers in the control group, PTOR mothers exhibited improved oral hygiene indicated by a lower Plaque Index (*p* < 0.05, Figure 2F) and a reduced dental caries condition, as indicated by fewer decayed teeth and surfaces (*p* < 0.05, Figures 2G,H).

**Figure 3:**
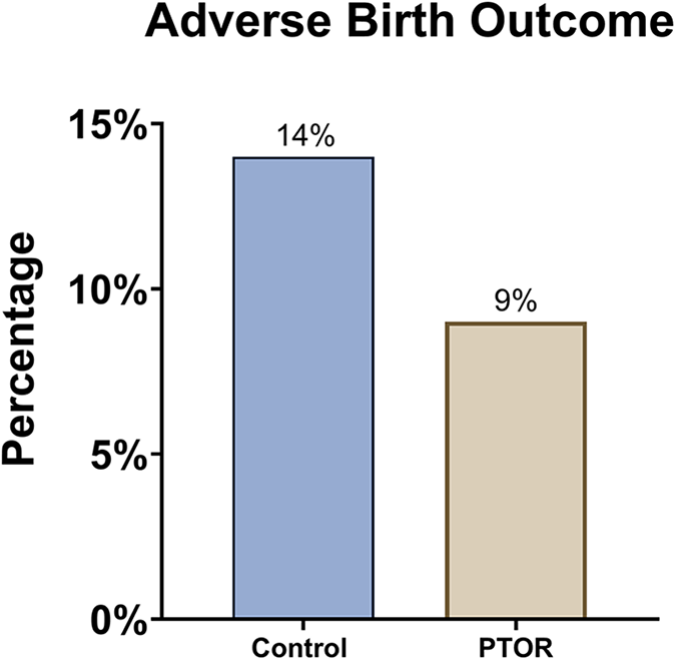
Incidence of adverse birth outcomes. Either preterm delivery before 37 gestational weeks or a birth weight lower than 2,500 g is considered an adverse birth outcome. The rate of adverse birth outcomes among infants born to mothers who received PTOR (1/11) was lower than that among the infants in the control group (3/22).

In addition, adverse birth outcomes (Figure 3) were observed in 9% of the PTOR group, which was lower than the 14% observed in the matched control group, although this difference did not reach statistical significance. We observed a trend of improvement in gestational age (GA) at birth for PTOR infants (GA, 38.71 ± 2.87 weeks) compared with control infants (GA, 38.5 ± 1.30 weeks), although this difference was not statistically significant (*p* = 0.78). Similarly, PTOR infants showed a trend of having a higher *Z*-score for birth weight (0.15 ± 1.50) than control infants (−0.21 ± 0.94), with a *p*-value of 0.56.

### 3.3 Child dental home establishment, oral health conditions, and microbial detection

**Figure 4:**
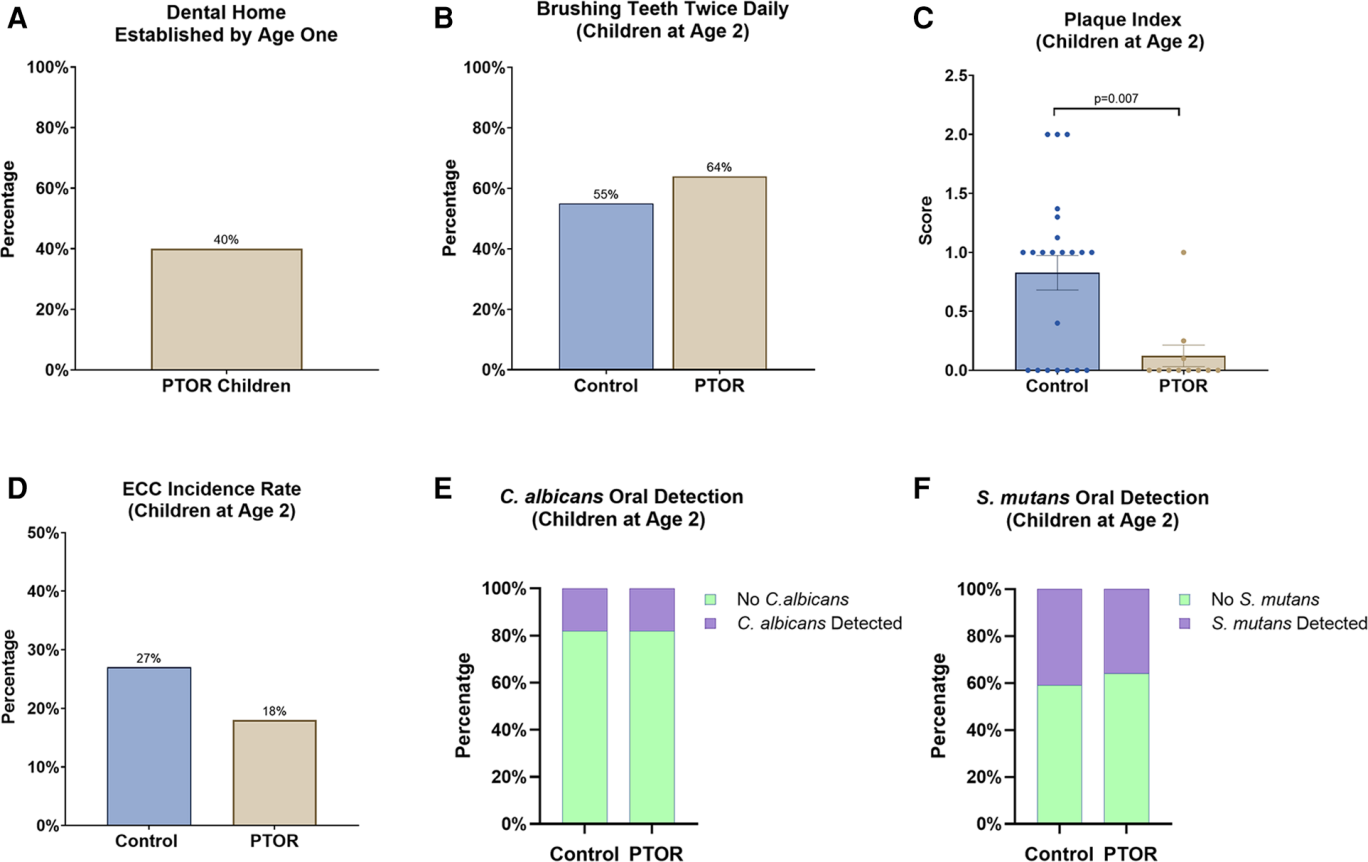
Dental care utilization, oral microbial carriage, and the oral health of children delivered by mothers receiving PTOR. (**A**) 40% of PTOR infants had a dental visit before 1 year of age, which is significantly higher than the national report for dental care utilization (less than 1% reported by Kolstad et al. in 2015). (**B**) A higher proportion of children in the PTOR group brushed teeth twice daily than in the control group. (**C**) A reduced Plaque Index, indicating better oral hygiene outcomes, was also found in the PTOR children's group compared with the control group (*p* = 0.007). (**D**) Children in the PTOR group had a lower rate of ECC onset than those in the control group, although no statistical difference was detected. (**E**) The oral detection of *C. albicans* from saliva and plaque samples showed no difference between the PTOR group (18%) and control group (18%). (**F**) 41% of children in the control group had *S. mutans* in their saliva and plaque samples compared with 36% in the PTOR group; this difference was not statistically significant.

Forty percent of children in the PTOR group had established a dental home by the age of 1 year (Figure 4A). Partly owing to the improved perinatal oral health literacy of the PTOR mothers, 64% of children in the PTOR group brushed their teeth twice daily (Figure 4B), compared with 55% of children in the control group. Subsequently, the Plaque Index decreased in the PTOR group compared with the control group (*p* < 0.05, Figure 4C). Regarding the caries status reflected by ECC onset by 2 years of age, ECC was detected in 2 out of 11 subjects in the PTOR group and in 6 out of 22 subjects in the control group (Figure 4D). Furthermore, the detection rate of *C. albicans* in saliva and plaque samples was not different between the PTOR (18%) and control (18%) groups (Figure 4E). In terms of *S. mutans*, 41% of the children in the control group had *S. mutans* in their saliva and plaque samples, compared with a detection rate of 36% in the PTOR group (Figure 4F).

## 4 Discussion

Poor maternal and child oral health has emerged as an increasingly acknowledged public health concern in the USA and globally, particularly affecting racially and socioeconomically disadvantaged populations (21, 24). Our study focused on an innovative total oral rehabilitation approach for underserved mothers during pregnancy and observed oral health benefits for mothers and their infants. Although this PTOR study is considered a pilot study and had a limited sample size, to the best of our knowledge, it is one of the few investigations assessing the impact of dental care regimens among underserved mothers and infants (9).

### 4.1 Impact of PTOR on mothers’ oral health

Several studies (1, 25) have highlighted caries risk parameters across the three trimesters and the postpartum period, revealing significant increases in salivary *S. mutans* during the second and third trimesters, as well as the postpartum period. In addition, mean pH and salivary total calcium content notably decreased during the third trimester and postpartum period (26). These shifts were reflected in the DMFT score, which exhibited an increase during the third trimester and postpartum period. Öztürk et al. concluded that the physiological conditions of pregnancy demonstrate both an initiating and accelerating effect on the cavitated lesions, spanning from the onset of pregnancy to the 6–8-week lactation period (1, 27).

Overall, the earlier studies have shown that preventive care measures in pregnant women or mothers with young children led to a reduction in *S. mutans* levels in mothers and a decrease in the colonization of these microorganisms, resulting in reduced caries development in their children (13, 28, 29). Our results highlighted that PTOR significantly decreased salivary and plaque *S. mutans* in the oral cavities of mothers up to the 1-year follow-up compared with the baseline (*p* < 0.05), which is consistent with the earlier study that implemented oral environmental stabilization, including atraumatic restorative treatment, demonstrating a substantial decrease in *S. mutans* with a short follow-up (1 week) (3). *S. mutans* typically adheres to the tooth surfaces and tends to accumulate at carious lesions; consequently, the restoration of cavitated teeth reduces the available binding sites for *S. mutans* in the oral cavity (30).

Our study observed a decrease in *S. mutans* levels at a 2-year follow-up, although it was not statistically significant compared with the baseline, suggesting a potential temporal trend in the microbial response to oral rehabilitation treatment interventions. This finding aligns with previous studies that have revealed that extensive operative treatment and extractions are believed to effectively lower levels of caries-associated microorganisms for up to 6 months (31). Similar findings were reported by Litsas, who observed the reappearance of *S. mutans* 3 months after full mouth rehabilitation in children with ECC (32). Twetman et al. noted no significant changes in *S. mutans* or Lactobacilli 5 months after the first post-treatment recall (33). Consequently, regular dental exams every 6 months are recommended to prevent the reappearance of cariogenic bacteria and further tooth decay, reducing the risk of future cavities and promoting long-term oral health.

PTOR did not have a similar impact on salivary *C. albicans*, which infiltrates mucosal surfaces other than tooth surfaces (34, 35). Our results up to 2 years of follow-up demonstrated that there was a noticeable reduction in salivary and plaque *C. albicans*; however, the difference between the baseline and follow-up visits did not reach statistical significance (*p* > 0.05). Interestingly, a significantly lower amount of plaque *S. mutans* and salivary and plaque *C. albicans* was observed among the PTOR mothers 2 years after childbirth, compared with the mothers in the control group (*P* < 0.05). This result further indicates the benefit of PTOR, with participants who underwent PTOR experiencing a reduction in oral pathogen carriage compared with those who did not receive PTOR.

### 4.2 The impact of PTOR on perinatal oral healthcare literacy and dental utilization

The PTOR study showed promising results, indicating a consistent improvement in perinatal oral healthcare literacy after the implementation of PTOR, with a statistically significant difference observed between the baseline and the assessment conducted up to 2 years postpartum (*p* < 0.05). This progress is particularly critical considering the well-established connection between the oral health literacy of caregivers and dental utilization of mothers and their children. Our results revealed a significant improvement in dental utilization over the past 12 months at both 1 and 2 years postpartum among women who received PTOR, which consequently reduced the incidence of dental emergencies. Our findings align with existing reports indicating a low dentist visit rate of 53% among low-income pregnant women, emphasizing their limited awareness of caries prevention and oral healthcare. Notably, 40% of children in the PTOR group had established a dental home by the age of 1 year, a significantly higher proportion than the 1% reported in a previous study conducted in a US region (36). In addition, Grembowski et al. demonstrated that improving mothers’ access to dental care for young Medicaid-enrolled children could positively impact the children's utilization of dental and preventive services, potentially alleviating racial and ethnic disparities in oral health (37). Addressing this knowledge gap becomes imperative, and our results suggest that improving perinatal oral health literacy in this demographic is achievable through targeted education and practical experiences, as evidenced by the positive impact of PTOR.

### 4.3 Impact of PTOR on children oral health and behaviors

According to the Fisher-Owens conceptual model of child oral health, the mother plays a crucial role in influencing the child's oral health, considering both biological and environmental factors. This correlation is likely a consequence of the considerable impact of maternal oral health and behaviors on child oral health (16), along with the well-established transmission of oral microorganisms from mothers to infants through vertical transmission (2, 3, 11, 19). Previous studies have shown that 70% of mothers and children have genetically identical *S. mutans* strains (38), and one of our previous studies indicated that over 60% of mothers and preschool children share genetically identical strains of the early colonizer *C. albicans* (39). A healthier maternal oral health is associated with a reduced likelihood of dental issues in the baby (40).

A previous study involving economically disadvantaged African-American mothers and their children identified a correlation between mothers’ bedtime tooth brushing habits and the dental care routines of their preschool-aged children (41), which is consistent with our study's results that revealed improved oral hygiene practices among infants with PTOR, specifically regarding tooth brushing frequency. Notably, 64% of children in the PTOR group brushed their teeth twice daily, a significant increase compared with 55% in the control group. This disparity in tooth brushing frequency was further reflected by the reduction in the Plaque Index, demonstrating statistical significance (*p* < 0.05) when compared with the control group.

In an earlier study conducted by Gunay et al. in 1998 in Germany, prenatal oral healthcare was found to reduce ECC and lower *S. mutans* carriage in children; notably, 100% of the children born to mothers who received prenatal care remained *S. mutans*-free up to 3 years of age in contrast to the control group (42). In addition, children of mothers who underwent prenatal oral healthcare had significantly lower salivary *S. mutans* levels (29). These findings supported the implementation of a proactive oral health program during pregnancy, which has been shown to effectively reduce the prevalence of severe ECC (43). This finding is supported by other research demonstrating a significant association between poor maternal oral health and ECC prevalence in children, regardless of poverty status (12). Studies conducted by Reisine et al. for black mothers in Detroit and Weintraub et al. (44) for Hispanic people in a rural area of California further supported this association (45). In our study, we observed a lower ECC incidence (18%) among the PTOR children than among the control group (27%), but this difference was not statistically significant, primarily due to the limited sample size. In addition, it is noteworthy that there was no significant difference in the oral detection rates of *S. mutans* and *C. albicans* between the PTOR group and the control group among 2-year-old children. This result is potentially due to the limited same size and the impact of potential non-maternal factors, such as dietary habits and environmental contacts on oral microbial colonization in early infancy (46). Further clinical studies with large sample sizes are warranted to elucidate the role of various factors in the establishment of oral microbiota during early childhood. In addition, future large-scale clinical trials are needed to validate our initial findings.

### 4.4 Limitations

When considering the results of our study, it is essential to recognize the following limitations: (1) The sample size was limited. (2) The study was restricted to a single city in the USA, which makes generalizing to other populations unreliable, particularly due to the small and convenient sample size. (3) The control group consisted of historical data from a separate study despite using propensity scores to match the control and treatment groups.

### 4.5 Implications and future directions

Promising strategies for preventing ECC encompass a range of interventions aimed at various stages, including prenatal and postnatal care, preventive dental programs for pregnant women, dietary advice, and prenatal oral healthcare along with increasing oral healthcare knowledge during pregnancy. Timely oral healthcare education and dental services, especially for first-time pregnant women in their first trimester, are recommended to prevent dental issues in subsequent trimesters and the postpartum period when dental treatment can be challenging.

Future studies with larger sample sizes and diverse populations are warranted to further evaluate the effectiveness of these preventive measures in reducing ECC prevalence. Healthcare providers should identify high-risk women for dental caries, ideally before pregnancy, and collaborate with dentists and hygienists. Encouraging pregnant women to schedule dental visits at their first prenatal appointment and follow oral hygiene recommendations is crucial. In addition, policymakers are encouraged to improve maternal and child oral health programs to integrate oral health into maternal health service and address disparities in dental care access and research.

## 5 Conclusions

PTOR is associated with sustained oral health benefits for mothers and significantly improves dental care utilization for mothers and their infants. Large-scale clinical trials are warranted to validate these study findings.

## Data Availability

The raw data supporting the conclusions of this article will be made available by the authors, without undue reservation.
