# Supplementary material for: Maternal and infant oral health benefits from mothers receiving prenatal total oral rehabilitation: a pilot prospective birth cohort study
Source: Front Oral Health. 2024 Aug 13;5:1443337. doi: 10.3389/froh.2024.1443337 (PMC11347430; doi:10.3389/froh.2024.1443337)

Study: Effect of Prenatal Oral Appendix Health Care on Oral Microbial Community – a Pilot Study

Subject ID ______ Date _______ Examiner (Initials) _________

Form: Demographic-Medical background (Mother)

Name ________________________ **Birth date**________________ **Sex:** F M

Race: American Indian/Alaska Native Asian Native Hawaiian or Pacific Islander Black or African American

Caucasian More than one race unkown or unreported

Ethinicity: Hispanic Non-Hispanic

If minor, parents names ______________________________ Relation to the study subject ______________________________

Home phone _____________ Cell phone _______________ Email address ____

Mailing address ____________________________________ City ____________________ State ________ Zip ___________

MEDICAL HISTORY

Does study subject has or had any of the following? (Please check any that apply)

- ❑ Cancer or tumor
- ❑ Heart ailment or angina
- ❑ Heart murmur, mitral valve prolapse, heart defect
- ❑ Rheumatic fever or rheumatic heart disease
- ❑ Artificial joint or valve
- ❑ High or low blood pressure
- ❑ Pacemaker
- ❑ Tuberculosis or other lung problems
- ❑ Kidney disease
- ❑ Hepatitis or other liver disease
- ❑ Alcoholism
- ❑ Blood transfusion
- ❑ Diabetes
- ❑ Neurologic condition
- ❑ Epilepsy, seizures, or fainting spells
- ❑ Emotional condition
- ❑ Arthritis
- ❑ Herpes or cold sores
- ❑ AIDS or HIV positive
- ❑ Migraine headaches or frequent headaches
- ❑ Anemia or blood disorders
- ❑ Abnormal bleeding after extractions, surgery, or trauma
- ❑ Hayfever or sinus trouble
- ❑ Allergies or hives
- ❑ Asthma
- ❑ **NONE OF ABOVE**

Allergies (Please check any that apply)

- ❑ Latex materials
- ❑ Penicillin or other antibiotics
- ❑ Local anesthetics ("Novocain")
- ❑ Codeine or other narcotics
- ❑ Sulfa drugs
- ❑ Barbiturates, sedatives, or sleeping pills
- ❑ Aspirin
- ❑ Other:________________________________
- ❑ **NONE OF ABOVE**

Medications (Please check any that apply)

- ❑ Aspirin
- ❑ Anticoagulants (blood thinners)
- ❑ Antibiotics or sulfa drugs
- ❑ High blood pressure medicine
- ❑ Antidepressants or tranquilizers
- ❑ Insulin, Orinase,s or other diabetes drug
- ❑ Nitroglycerin
- ❑ Cortisone or other steroids
- ❑ Osteoporosis (bone density) medicine
- ❑ Other:________________________________
- ❑ **NONE OF ABOVE**

Smoke or use chewing tobacco?

- ❑ Yes
- ❑ No

Study: Effect of Prenatal Oral Health Care on Oral Microbial Community – a Pilot Study

Subject ID_________ Date________ Examiner (initials)_____

Form: Demographic and Oral hygiene practice questionnaire (Mother)

Please read each question carefully. Fill in the blank or place a check mark (✓) in the BOX next to the answer that best describes you.

Part 1: Social-demographic background

1. What is your current work status?
   - ☐1 I am currently employed
   - ☐2 I am currently unemployed

2. If currently employed, what is your most recent occupation?

   ____

3. What is the highest grade you completed?
   - ☐1 Middle school
   - ☐2 High school
   - ☐3 More than High School
   - ☐4 College level
   - ☐5 Postgraduate level

4. What is your marital status?
   - ☐1 Married
   - ☐2 Single
   - ☐3 Separated
   - ☐4 Divorced
   - ☐5 Widowed
   - ☐6 Other

Part 2: History of yeast infection

5. Do you have history of yeast infection?
   - ☐1 No
   - ☐2 Athlete's foot
   - ☐3 Ringworm
   - ☐4 Cradle cap (when you were a child)
   - ☐5 Oral thrush
   - ☐6 Vaginal thrush
   - ☐7 Angular stomatitis
   - ☐8 Systemic candidiasis
   - ☐9 other: ______________

6. Have you had long term (>3month) antibiotics use?

- ☐1 No
- ☐2 Yes, please specify ____________________

7. Have you had antifungal therapy (treating yeast infection) in the past 3 month?

- ☐1 No
- ☐2 Yes, please specify ___________________

Part 3: Hygiene

8. How often do you brush teeth?

- ☐1 Twice/daily
- ☐2 Once/daily
- ☐3 Not everyday
- ☐4 Never

9. In the last 12 months did you have a toothache or injury that needed care right away in the emergency room or dental urgent care?

- ☐0 Yes
- ☐1 No

10. When was the last time you had a dental check-up?

- ☐1 In the past 12 months
- ☐2 1 to 2 years ago
- ☐3 More than 2 years ago
- ☐4 Never

Subject ID_________ Date________ Examiner (initials)_____

Form: Perinatal oral health care literacy (Mother)

1. Cleaning baby teeth is not important because they fall out anyway
   1) Agree (0)
   2) Disagree (1)
   3) Don't know (0)

2. A child's overall health does not depend on whether he or she has cavities in baby teeth
   1) Agree (0)
   2) Disagree (1)
   3) Don't know (0)

3. A cavity in a baby tooth should be filled only when it hurts
   1) Agree (0)
   2) Disagree (1)
   3) Don't know (0)

4. Fluoride helps prevent tooth decay
   1) Agree (1)
   2) Disagree (0)
   3) Don't know (0)

5. It safe for me to drink water that has fluoride
   1) Agree (1)
   2) Disagree (0)
   3) Don't know (0)

6. Tooth decay in baby teeth can cause infections that can spread to the face and other parts of the body
   1) Agree (1)
   2) Disagree (0)
   3) Don't know (0)

7. The germs in my mouth can be passed to my child if I kiss her/him on the lip, taste food for her/him, or share utensils.
   1) Agree (1)
   2) Disagree (0)
   3) Don't know (0)

Study: Effect of Prenatal Oral Health Care on Oral Microbial Community – a Pilot Study

Subject ID_________ Date________ Examiner (initials)_____

Form: Numeric Rating Scale (NRS) (Mother)

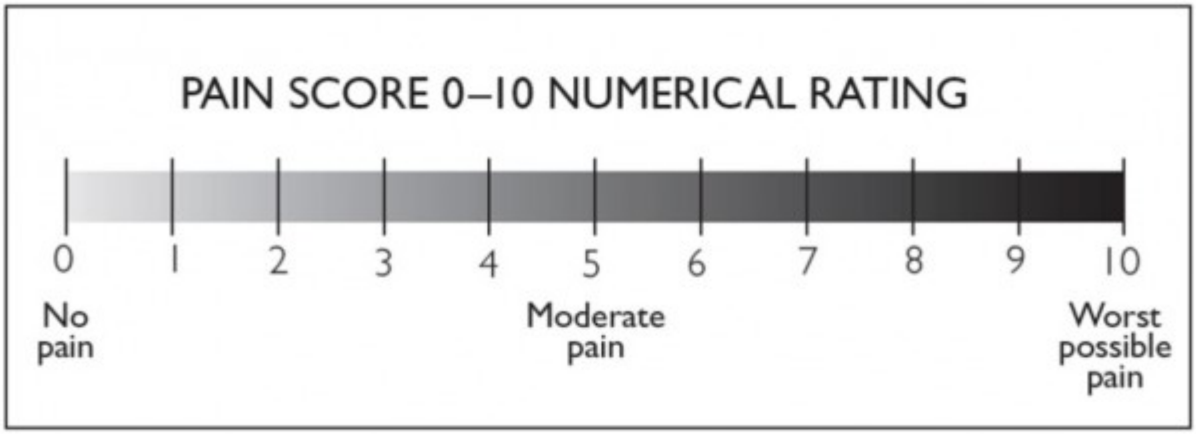

Supplement: Supplementary file 1 [file Datasheet1.pdf]
