# Supplementary material for: Maternal and infant oral health benefits from mothers receiving prenatal total oral rehabilitation: a pilot prospective birth cohort study
Source: Front Oral Health. 2024 Aug 13;5:1443337. doi: 10.3389/froh.2024.1443337 (PMC11347430; doi:10.3389/froh.2024.1443337)
Supplement: Supplementary file 2 [file Datasheet2.pdf]

**Study: Effect of Prenatal Oral Health Care on Oral Microbial Community – a Pilot Study**

**Subject ID ______ Date _______ Examiner (Initials) _________**

# Form: Demographic-Medical background (Child)

**Name** ________________________ **Birth date**________________ **Sex:** F M

**Race:** American Indian/Alaska Native Asian Native Hawaiian or Pacific Islander Black or African American

Caucasian More than one race Unknown or unreported

**Ethnicity:** Hispanic Non-Hispanic

If minor, parents names __________________ Relation to the study subject ___________________________________

Home phone _____________ Cell phone _______________ Email address __________________________________

Mailing address _____________________________ City ____________________ State ________ Zip ___________

## MEDICAL HISTORY

**Does study subject has or had any of the following?**
**(Please check any that apply)**

- ❑ Cancer or tumor
- ❑ Heart ailment or angina
- ❑ Heart murmur, mitral valve prolapse, heart defect
- ❑ Rheumatic fever or rheumatic heart disease
- ❑ Artificial joint or valve
- ❑ High or low blood pressure
- ❑ Pacemaker
- ❑ Tuberculosis or other lung problems
- ❑ Kidney disease
- ❑ Hepatitis or other liver disease
- ❑ Alcoholism
- ❑ Blood transfusion
- ❑ Diabetes
- ❑ Neurologic condition
- ❑ Epilepsy, seizures, or fainting spells
- ❑ Emotional condition
- ❑ Arthritis
- ❑ Herpes or cold sores
- ❑ AIDS or HIV positive
- ❑ Migraine headaches or frequent headaches
- ❑ Anemia or blood disorders
- ❑ Abnormal bleeding after extractions, surgery, or trauma
- ❑ Hayfever or sinus trouble
- ❑ Allergies or hives
- ❑ Asthma
- ❑ **NONE OF ABOVE**

**Allergies (Please check any that apply)**

- ❑ Latex materials
- ❑ Penicillin or other antibiotics
- ❑ Local anesthetics ("Novocain")
- ❑ Codeine or other narcotics
- ❑ Sulfa drugs
- ❑ Barbiturates, sedatives, or sleeping pills
- ❑ Aspirin
- ❑ Other:_______________________________________
- ❑ **NONE OF ABOVE**

**Medications (Please check any that apply)**

- ❑ Aspirin
- ❑ Anticoagulants (blood thinners)
- ❑ Antibiotics or sulfa drugs
- ❑ High blood pressure medicine
- ❑ Antidepressants or tranquilizers
- ❑ Insulin, Orinase,s or other diabetes drug
- ❑ Nitroglycerin
- ❑ Cortisone or other steroids
- ❑ Osteoporosis (bone density) medicine
- ❑ Other:_____________________________________
- ❑ **NONE OF ABOVE**

**Study: Effect of Prenatal Oral Health Care on Oral Microbial Community – a Pilot Study**

**Subject ID_________ Date________ Examiner (initials)_____**

# Form: Questionnaire for Child

**Please read each question carefully. Fill in the blank or place a check mark (✓) in the BOX next to the answer that best describes you.**

***Part 1: Delivery method, hygiene and childcare.***

1. How much did your child weigh when he/she was born?

   ________

2. By which method was your child delivered?
   - ☐1 Vaginal
   - ☐2 C-section

3. How often do you brush your child's teeth?
   - ☐1 Twice/daily
   - ☐2 Once/daily
   - ☐3 Not everyday
   - ☐4 Never
   - ☐5 My child doesn't have erupted tooth (teeth)

4. Who is your child's direct care provider at home?
   - ☐1 Mom
   - ☐2 Dad
   - ☐3 Grandmother
   - ☐4 Grandfather
   - ☐5 Others___________

5. Does your child attend daycare center?
   - ☐1 Yes, part time
   - ☐2 Yes, full time
   - ☐3 No

***Part 2: Yeast infection***

6. Does your child have history of yeast infection?
   - ☐1 No
   - ☐2 Athlete's foot
   - ☐3 Ringworm
   - ☐4 Cradle cap
   - ☐5 Oral thrush

☐6 Systemic candidiasis
☐7 other: ______________

7. Has your child had long term (>3month) antibiotics use?
   ☐1 No
   ☐2 Yes, please specify ____________________

8. Has your child had antifungal therapy (treating yeast infection) in the past 3 month?
   ☐1 No
   ☐2 Yes, please specify ___________________

***Part 3: Feeding method***

9. What did you feed your child in the first 3 months? (Choose all that apply)
   ☐1 Breast feeding (go to question 10 if you choose this answer)
   ☐2 Bottle feeding (go to question 11 if you choose this answer)

10. How often were your child breast feed at night? (If applicable)
    ☐1 More than once a night
    ☐2 Once every night
    ☐3 Several times a week
    ☐4 Several times a month
    ☐5 Never

11. How often did your child have milk formula at night? (if applicable)

    ☐1 More than once a night
    ☐2 Once every night
    ☐3 Several times a week
    ☐4 Several times a month
    ☐5 Never

---

**THANK YOU FOR ANSWERING THESE QUESTIONS**

**Please return the completed questionnaire to the study staff**
